# Association between systemic inflammatory response index and eczema among children and adolescents: a cross-sectional study based on NHANES database

**DOI:** 10.3389/fped.2025.1532765

**Published:** 2025-05-16

**Authors:** Tianping Song, Yueying Dai, Kaiyuan Xue, Suqing Yang, Rui Yuan

**Affiliations:** ^1^ Department of Graduate, School of Heilongjiang University of Chinese Medicine, Harbin, China; ^2^ Department of Dermatology, The First Affiliated Hospital, Heilongjiang University of Chinese Medicine, Harbin, China

**Keywords:** systemic inflammatory response index, eczema, NHANES, association, a cross-sectional study

## Abstract

**Background:**

In previous studies, the systemic inflammatory response index (SIRI) might be a predictor for chronic inflammation, but the relationship between SIRI and eczema continues to be ambiguous. The objective of the study was to clarify the connection between the level of SIRI and eczema prevalence among children and adolescents.

**Methods:**

The National Health and Nutrition Examination Survey (NHANES) was the database from which we accessed information, comprising participants aged 3–19 years. Furthermore, the investigation of the association between SIRI and eczema was carried out by using logistic regression, and restricted cubic spline models were used to explore nonlinear relationships.

**Results:**

A total of 3,397 subjects, featuring a median age of 11.97 ± 4.87 years, were selected, and 368 (10.83%) were diagnosed with eczema among these participants. Statistically significant differences were observed in the baseline SIRI characteristics for age, race, and BMI quartiles (*p* < 0.001). In adjusted logistic regression models, the negative association between SIRI and eczema was indicated (OR: 0.83; 95% CI: 0.69–1.00, *p* < 0.05), suggesting that a one-unit increase in SIRI corresponds to a 17.17% decline in the odds of eczema prevalence. Meanwhile, a nonlinear relationship was revealed by the restricted cubic spline (RCS) between SIRI and eczema prevalence among children and adolescents. The findings of subgroup analysis suggested that there were no significant effects of any covariates on this relationship (all *p* for interaction > 0.05).

**Conclusion:**

The association between SIRI and eczema prevalence in children and adolescents is negative, indicating that elevated SIRI exhibits a protective effect against eczema in children and adolescents, whereas those with low SIRI may require closer monitoring for eczema development.

## 1 Introduction

Eczema is an inflammation of the skin condition that impacts the superficial dermis and epidermis, which is influenced by various factors (1). It is marked by dysfunction in the skin barrier and abnormally hyperactive immune responses, resulting in symptoms such as itchy rashes and dry skin (2), which can progress with age (3, 4). A study shows that the proportion of children affected by eczema can vary from 10.6% to 35.7% (5). Scratching can trigger a harmful cycle of skin damage, leading to abrasions and increased inflammation (6). Furthermore, eczema is involved with a breadth of comorbid conditions, including depression, anxiety, and sleep issues (7–10), all of which can severely impair a child's quality of life (11, 12) and create considerable financial strain for families (13). A significant aspect of eczema is the compromised function of the skin barrier (14). Reduction of critical proteins and lipids comprising the stratum corneum compromises the skin barrier function, enabling the penetration of irritants, including allergens and microbes, which may subsequently exacerbate tissue damage (15). Immune dysfunction is essential to the disease's development, noted for T helper type 2 (Th2) cells' activation and the cytokines' release like interleukin-4 (IL-4), interleukin-5 (IL-5), interleukin-9 (IL-9), and interleukin-13 (IL-13), contributing to itching, inflammation, and tissue damage (16, 17). Disruptions in the skin's microbial balance can worsen inflammation and further damage the skin barrier, often leading to an overgrowth of Staphylococcus aureus (18, 19). Environmental factors, such as changes in climate and exposure to allergens like dust mites, pollen, and pollutants, can exacerbate eczema symptoms and perpetuate inflammation (20). Genetic influences are significant in determining an individual's risk of eczema, as certain variants impact protective epidermal shield integrity, immune system modulation, and the differentiation of epidermal cells, thereby heightening the likelihood of progression to atopic dermatitis (AD) (21). While no curative treatment currently exists for eczema, ongoing investigations into novel risk factors and biomarkers hold promise for advancing assessment protocols and preventive approaches, underscoring the need to elucidate the multifactorial etiology of this condition.

Researches have indicated that eczema is a chronic inflammatory skin disorder, and evaluating circulating pro-inflammatory markers is fundamental to diagnosing and determining the prognosis of different chronic diseases (22, 23). The systemic inflammatory response index (SIRI) is an innovative inflammatory marker that incorporates immune cell subsets (24). It captures the balance between systemic inflammation and the immune response through neutrophils, lymphocytes, and monocytes, reflecting the overall systemic inflammatory state (25). The evaluation of SIRI is straightforward, relying on the counts of these three blood cell types. It is increasingly utilized to explore the connections between chronic inflammation and a range of diseases, such as metabolic disorders, cancer, and other inflammatory conditions (23, 26, 27). In a retrospective study, the findings indicated that SIRI surpassed platelet-to-lymphocyte ratio (PLR), neutrophil-to-lymphocyte ratio (NLR), and systemic immune-inflammation index (SII), and demonstrated potential benefits for diagnostic and prognostic indicators for Bell's palsy (28). The association between the elevation of SIRI levels and the reduction of patients' overall survival (OS) with breast cancer was demonstrated in a meta-analysis (29). In another retrospective clinical study focusing on patients suffering from distal cholangiocarcinoma (dCCA) who were treated with pancreatoduodenectomy (PD), the survival analysis indicated that the lower SIRI levels, the better prognoses with statistical significance (*p* < 0.001), supporting the conclusion that SIRI serves as a reliable and independent indicator for recurrence-free survival (RFS) and 5-year OS for prognosis (30). The prospect of SII and SIRI as capable diagnostic devices for gestational diabetes mellitus (GDM) was highlighted in a recent study. The findings indicated that elevated levels of SII and SIRI in early pregnancy were associated with an amplified probability of GDM's initial stages (31).

However, the exact dynamics of the relationship between SIRI and eczema remain incompletely understood, and its prognostic potential in this disease context has yet to be firmly established. In order to investigate this latent relationship, the authors turned to the National Health and Nutrition Examination Survey (NHANES) database, seeking to provide new perspectives on eczema prevention and treatment. The authors hypothesized that SIRI could predict the prevalence of eczema in pediatric populations.

## 2 Methods

### 2.1 Data sources

NHANES is a multi-stage, ongoing cross-sectional survey, the purpose of which is to evaluate the American's health and nutrition status. NHANES is a significant undertaking of the National Center for Health Statistics (NCHS), which functions under the Centers for Disease Control and Prevention (CDC) and is assigned to compile critical health data for the country. The NHANES survey has been granted permission by the NCHS Research Ethics Review Board, and all survey participants, as well as the parents or legal guardians of those under 16 years, have given written informed consent.

### 2.2 Study population

**Figure 1:**
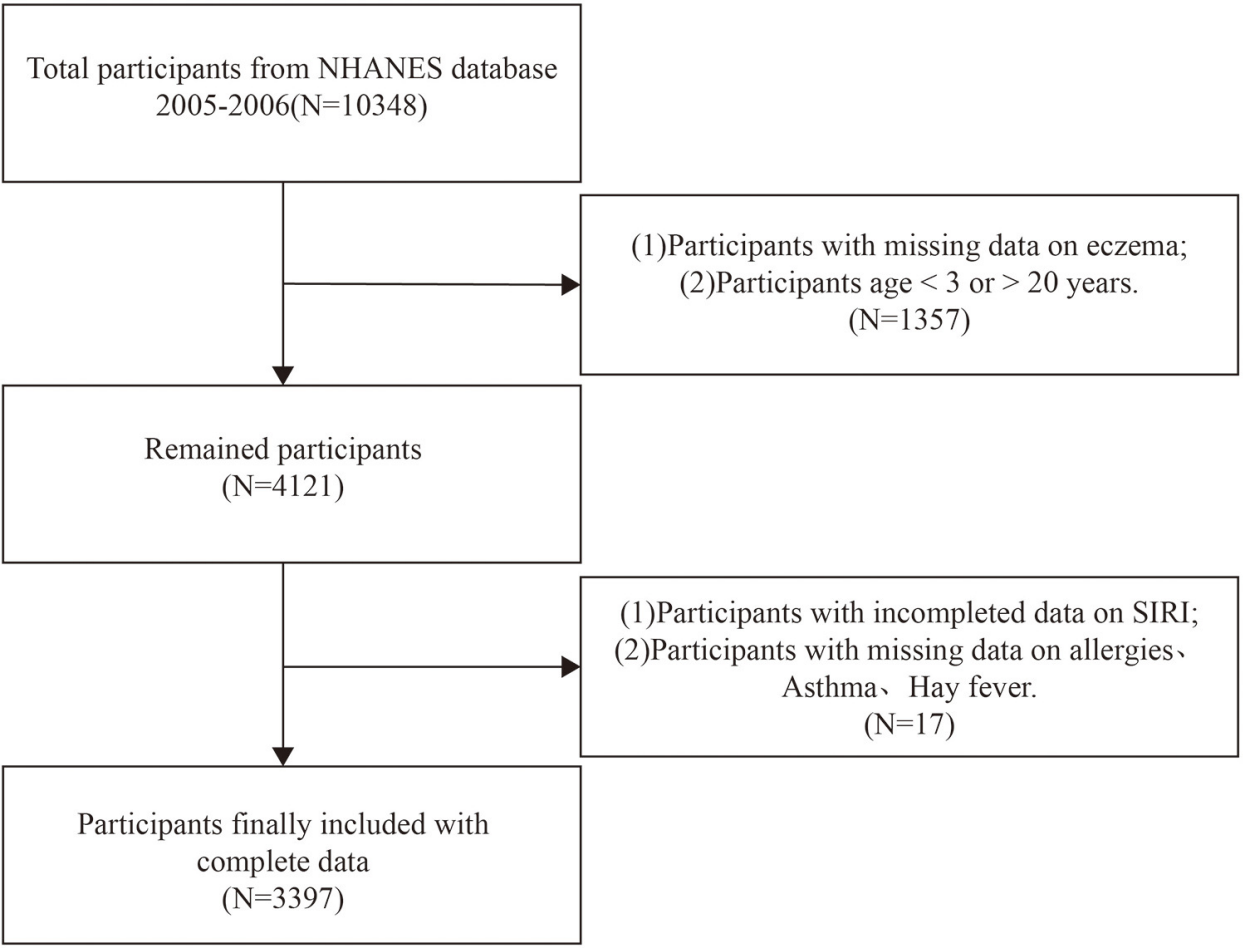
Flow chart of participants selection.

In this cross-sectional investigation, we used NHANES survey data from the 2005–2006 cycle, as it is the only cycle providing comprehensive questionnaire data related to allergies, relevant to eczema as an outcome variable. Initially, 10,348 participants were considered. The exclusion criteria were: (1) lack of data on eczema diagnosis, (2) age < 3 years or >19 years, (3) missing data on SIRI, and (4) absence of data on other covariates. The screening process is illustrated in Figure 1.

### 2.3 Exposure variable and outcome variable

SIRI is an emerging biomarker and predictor of inflammation. The Mobile Examination Center (MEC) is the place where the medical staff collect peripheral blood samples from NHANES participants with the tool of a Beckman Coulter HMX blood analyzer. A complete blood count was conducted to measure lymphocytes, neutrophils, and monocytes. SIRI levels were calculated as follows: (monocyte count × neutrophil count)/lymphocyte count, with counts and results reported in units of 10³ cells/ml (32). SIRI served as the exposure variable in this study.

The definition of eczema in this study depends on the question, “Has a doctor or other health professional ever told you that you have eczema (ek-zi-ma)?” (https://wwwn.cdc.gov/Nchs/Data/Nhanes/Public/2005/DataFiles/AGQ_D.htmAGQ_D) Participants who either declined to answer or responded with “don't know” were excluded from the analysis. The question was asked before the physical examination, in the home, using the Computer-Assisted Personal Interviewing (interviewer-administered) (CAPI) system. This study used the presence or absence of eczema as the outcome variable.

### 2.4 Covariates

This study investigated potential confounding factors that could impact eczema. The covariates selected for inclusion were based on their relevance to eczema and the inclusion of similar studies in the past. The covariates included demographic variables [sex, age, race, family poverty income ratio (PIR)], body mass index (BMI, kg/m^2^), serum total immunoglobulin E (IgE) antibodies (kU/L), hay fever, allergies, and asthma.

Racial categories were classified into four groups: Mexican American, other Hispanic, non-Hispanic white, non-Hispanic black, and other races. The household poverty income ratio (PIR) was segmented into three groups based on the United States Department of Agriculture (USDA) food assistance program's eligibility criteria: low-income level (0.00–1.85), medium-income level (1.85–3.50), and high-income level (3.50+) (33). According to the guidelines proposed by the World Health Organization (WHO) in 2008 (34), BMI is grouped into three categories: normal weight (BMI < 25 kg/m^2^), overweight (25 kg/m^2^ ≤ BMI < 30 kg/m^2^), and obese (BMI ≥ 30 kg/m^2^). Serum total IgE antibodies are classified into low-level groups (<100 kU/L) and high-level groups (≥100 kU/L).

The medical comorbidities assessed as potential risk factors for eczema in this study included hay fever, allergies, and asthma. Participants who responded “yes” to these questions were classified as “ever had”, while those who indicated “no” or “don't know” were categorized as “never had”.

### 2.5 Statistical analysis

In this research, the NHANES database was applied. Continuous variables were calculated and displayed as means and standard deviations (SD), and categorical variables were expressed as numbers and percentages [(*n*)%]. *T*-tests and Chi-Square tests were respectively utilized to analyze continuous and categorical variables, and to examine the association between SIRI and eczema, multivariate linear regression was conducted with SIRI scores treated as a continuous variable, and three distinct models were subjected to logistic regression to analyze the association between SIRI scores and eczema, with the results presented as odds ratios (ORs) and 95% confidence intervals (CIs). In Model 1, no adjustments were applied to the variables. Adjustments for sex, age, race, and PIR were applied in Model 2. Every covariate, encompassing sex, age, race, PIR, BMI, serum total IgE, hay fever, allergies, and asthma, is accounted for in Model 3. We also used restricted cubic splines(RCS) to detect the nonlinear effect of SIRI on eczema. For the subgroup analysis, stratified factors including sex, age, race, PIR, BMI, serum total IgE, hay fever, allergies, and asthma were utilized as potential effect modifiers. To control for Type I errors, we implemented false discovery rate (FDR) correction for multiple hypothesis testing in subgroup analysis. We set the significance threshold at α = 0.05 and calculated FDR-adjusted *q-values* using the Benjamini-Hochberg (BH) method. In the process of handling missing data, we used multiple imputation to improve data integrity and the reliability of the analysis results, with skewed distributions filled in using the median and normally distributed data using the mean. All data analyses were performed using R software version 4.2.1 and Empower Stats (version 2.0), and *p* < 0.05 was considered statistically significant.

## 3 Results

### 3.1 Baseline characteristics of participants

A total of 3,397 participants ranging from 3 to 19 years of age were analyzed from the 2005–2006 NHANES database, the selected subjects featuring a median age of 11.97 ± 4.87 years. This sample comprised 1,686 boys (49.63%) and 1,711 girls (50.37%). Among these participants, 368 (10.83%) were diagnosed with eczema. Significant statistical differences (*p* < 0.001) were noted in baseline SIRI characteristics based on age, race, and BMI quartiles. A statistical difference (*p* < 0.05) was ascertained concerning the baseline SIRI characteristics on the subject of “whether or not you have ever had allergies”. The group with the highest SIRI showed both increased BMI and a higher prevalence of allergies, relative to the lowest SIRI group. The baseline characteristics are illustrated in Table 1.

**Table 1:** Basic characteristics of participants by systemic inflammation response index among children and adolescents.

Characteristics	Systemic inflammation response index				*p*-value
	Q1 (*N* = 827)	Q2 (*N* = 869)	Q3 (*N* = 841)	Q4 (*N* = 860)	
Age (years)	9.88 ± 4.83	11.43 ± 4.77	12.59 ± 4.61	13.90 ± 4.37	<0.001
Sex, (%)					0.195
Male	402 (48.61)	457 (52.59)	417 (49.58)	410 (47.67)	
Female	425 (51.39)	412 (47.41)	424 (50.42)	450 (52.33)	
Race/ethnicity, (%)					<0.001
Mexican American	199 (24.06)	273 (31.42)	296 (35.20)	369 (42.91)	
Other Hispanic	25 (3.02)	21 (2.42)	31 (3.69)	35 (4.07)	
Non-Hispanic White	152 (18.38)	236 (27.16)	268 (31.87)	226 (26.28)	
Non-Hispanic Black	399 (48.25)	284 (32.68)	207 (24.61)	188 (21.86)	
Other races	52 (6.29)	55 (6.33)	39 (4.64)	42 (4.88)	
Family PIR	2.05 ± 1.49	2.16 ± 1.56	2.12 ± 1.44	1.98 ± 1.44	0.057
BMI	18.99 ± 4.63	20.76 ± 5.77	22.57 ± 6.38	24.26 ± 6.96	<0.001
IgE (kU/L)	201.73 ± 446.84	204.57 ± 543.89	178.43 ± 371.38	182.99 ± 382.31	0.522
Allergies, (%)					0.039
Yes	176 (21.28)	215 (24.74)	195 (23.19)	233 (27.09)	
No	651 (78.72)	654 (75.26)	646 (76.81)	627 (72.91)	
Asthma, (%)					0.796
Yes	124 (14.99)	144 (16.57)	133 (15.81)	142 (16.51)	
No	703 (85.01)	725 (83.43)	708 (84.19)	718 (83.49)	
Hay fever, (%)					0.527
Yes	30 (3.63)	34 (3.91)	30 (3.57)	23 (2.67)	
No	797 (96.37)	835 (96.09)	811 (96.43)	837 (97.33)	
Eczema, (%)					<0.001
Yes	120 (14.51)	110 (12.66)	77 (9.16)	61 (7.09)	
No	707 (85.49)	759 (87.34)	764 (90.84)	799 (92.91)	

Mean ± SD for continuous variables: the *P* value was calculated by the weighted linear regression model; (%) for categorical variables: the *P* value was calculated by the weighted chi-square test. PIR, the ratio of income to poverty, BMI, body mass index; Q, quartile.

### 3.2 Analysis of the relationship between SIRI and eczema

Table 2 describes the relationship between SIRI and eczema. Current statistical results showed a correlation between higher levels of SIRI and a reduced risk of eczema (*p* < 0.05). This relationship was significant in the unadjusted model 1 (OR: 0.66; 95% CI: 0.55–0.80, *p* < 0.001). In model 2, which was adjusted for age, sex, race, and PIR, the association remained statistically significant (*p* < 0.05) with OR: 0.83, 95% CI: 0.69–0.99. In the fully adjusted model 3, SIRI still exhibited a negative correlation with eczema (OR: 0.83; 95% CI: 0.69–1.00, *p* < 0.05), suggesting that a one-unit increase in SIRI corresponds to a 17.17% decrease in the odds of eczema prevalence. This significant association persisted after performing a quartile analysis of SIRI, with model 3 results demonstrating that those in the top SIRI quartile (Q4) had a 32.49% reduction in eczema prevalence compared to individuals in the lowest quartile (Q1) (OR: 0.68; 95% CI: 0.47–0.97, *p* < 0.05).

**Table 2:** Associations between systemic inflammation response index and eczema among children and adolescents.

Exposure	Model 1 [β (95% CI)], *p* value	Model 2 [β (95% CI)], *p* value	Model 3 [β (95% CI)], *p* value
SIRI (continuous)	0.66 (0.55,0.80), <0.001	0.83 (0.69,0.99), 0.04	0.83 (0.69,1.00), 0.04
SIRI (quartile)			
Quartile 1	1 (ref)	1 (ref)	1 (ref)
Quartile 2	0.85 (0.65, 1.13), 0.27	0.91 (0.69, 1.22), 0.54	0.97 (0.72, 1.31), 0.84
Quartile 3	0.59 (0.44, 0.80), <0.001	0.67 (0.49, 0.92), 0.01	0.79 (0.57, 1.10), 0.16
Quartile 4	0.45 (0.33, 0.62), <0.001	0.58 (0.41, 0.81), 0.00	0.68 (0.47, 0.97), 0.03
*P* for tend	<0.001	<0.001	<0.001

Model 1: no covariates were adjusted. Model 2: age, sex, race, and PIR were adjusted. Model 3: age, sex, race, PIR, BMI, serum total IgE antibody, allergies, asthma, and hay fever were adjusted. PIR, the ratio of income to poverty, BMI, body mass index; Q, quartile; SIRI, systemic inflammatory response index.

**Figure 2:**
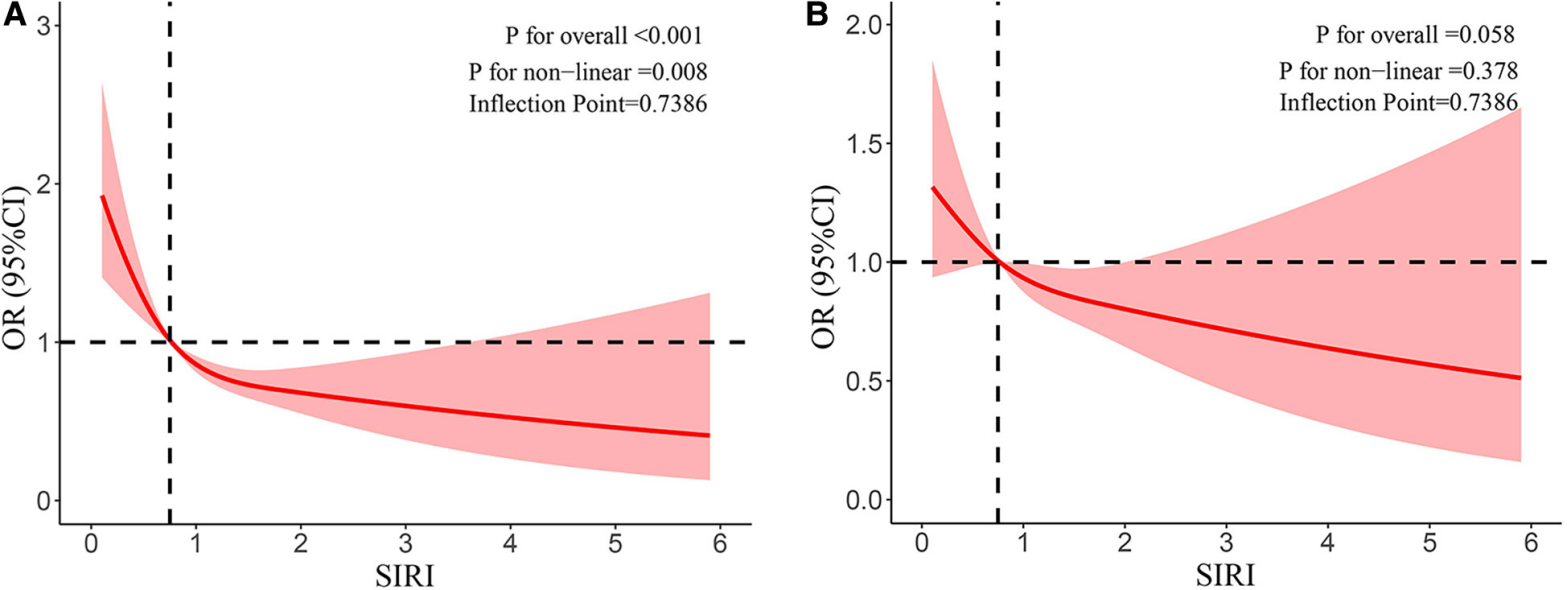
**(A)** Results of RCS after outlier values were removed (unadjusted model). **(B)** Results of RCS after outlier values were removed (adjusted model).

In addition, the negative association was reinforced by the restricted cubic splines between SIRI and eczema prevalence among children and adolescents. Figure 2 shows the results of the restricted cubic splines after outlier values were removed. The nonlinear association was observed in both unadjusted (Figure 2A) (*p* -value < 0.001, *p* -nonlinear < 0.05) and adjusted models (Figure 2B) (*p* –value > 0.05, *p* –nonlinear > 0.05).

### 3.3 Subgroup analysis

**Figure 3.**
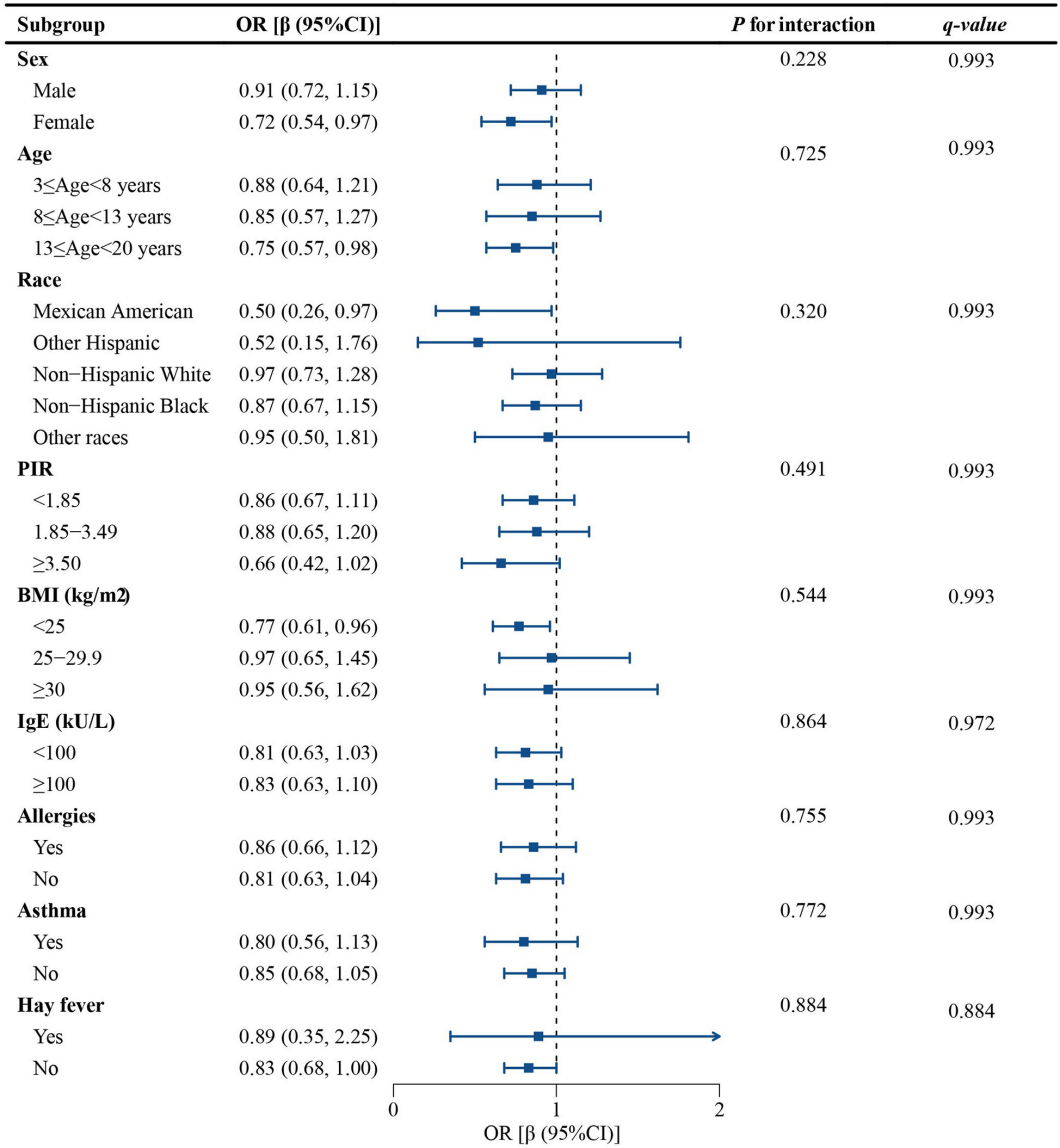
Results of subgroup analysis.

A subgroup analysis was conducted to determine whether the connection between SIRI and eczema remained consistent across various population groups. The findings suggested that there were no significant effects of any variables on this relationship (*p* for interaction all > 0.05). To control for Type I errors, we implemented FDR correction for multiple hypothesis testing. All *q-values* exceeded the threshold of *α* = 0.05, indicating no statistically significant findings. This multiple testing correction further reduced the risk of false positive results. As shown in Figure 3, all the stratification factors, such as sex, age, race, BMI, serum IgE antibodies, asthma, hay fever, and allergies, do not significantly affect the negative correlation between SIRI and eczema.

## 4 Discussion

This investigation employed a cross-sectional approach, involving 3,397 youth aged 3–19 from the 2005–2006 NHANES database. A negative correlation was found between SIRI and eczema prevalence, illustrating that an upsurge in SIRI levels is tied to a lower eczema prevalence in children and adolescents. No significant associations were identified between this correlation and covariates such as sex, age, race, serum IgE antibodies, BMI, asthma, hay fever, or allergies. Subgroup analysis and interaction testing confirmed that this association remained consistent across different subgroups.

To our knowledge, this study is the first investigation to analyze the connection between SIRI and eczema among children and adolescents. Systemic inflammation is a key characteristic of eczema, and routine blood tests, along with derived inflammatory indicators, are frequently employed to evaluate inflammatory conditions (17). Previous researches have explored the association between inflammatory markers from blood counts and a series of skin diseases. A clinical observation revealed a positive correlation between NLR and PLR concerning the activity of Bullous pemphigoid (BP) (35). SIRI and other blood count-derived inflammatory markers were recognized as effective indicators for assessing systemic inflammation and the severity of psoriasis (36). A cross-sectional study from NHANES also contributed to the findings that SIRI and psoriasis prevalence are positively associated (37). Additionally, a strong positive connection was observed between the psoriasis area severity index (PASI) and SIRI in a multicenter retrospective study, concluding that SIRI could be identified as an independent predictor for hyperresponse (38).

Potential underlying mechanisms for the negative association between SIRI and eczema in children and adolescents are summarized as follows. Firstly, the inverse correlation between SIRI and eczema prevalence may represent a protective effect conferred by relatively enhanced T helper type 1 (Th1) activity against the Th2-dominant pathophysiology of eczema. Th1 cells and Th2 cells represent two distinct subsets of T helper cells. During disease development, significantly overexpressed Th2 cells dominate the immune response (39). Earlier studies indicated that enhanced Th1 reactivity exerts protective effects against eczema development (40). The cytokine interferon-gamma (IFN-*γ*) produced by Th1 cells can inhibit Th2 cell differentiation, suppressing inflammatory responses (41). Therefore, individuals with elevated SIRI levels may exhibit relatively stronger Th1 activity. Secondly, individuals with low SIRI may exhibit impaired regulatory T (Treg) cells function. The fundamental immunoregulatory mechanism of Treg cells involves the secretion of inhibitory cytokines, like transforming growth factor-beta (TGF-β), attenuating inflammatory responses in eczema (42). Consequently, individuals with low SIRI may have insufficient Treg cell numbers or functional deficiency, which can lead to compromised immune tolerance, potentially resulting in compromised immune tolerance and subsequent exacerbation of eczema pathogenesis. Thirdly, SIRI levels may serve as an indicator of the host's immune reactivity to the cutaneous microbiome. In healthy skin, microbial diversity promotes balanced innate immune activation, resulting in moderate SIRI elevation that appears to confer protection against eczema development. Fourthly, the inverse correlation between SIRI and eczema prevalence observed in pediatric populations demonstrates marked age specificity. This phenomenon may stem from the ongoing dynamic development of the immune system during childhood and adolescence. The establishment of cutaneous microbiota during early life remains incomplete, elevated SIRI levels may indicate enhanced microbial recognition capacity. Finally, distinct dietary patterns may influence the SIRI, with pro-inflammatory diets potentially elevating eczema risk through the induction of systemic low-grade inflammation. Our findings may suggest that increasing intake of whole grains and vegetables while reducing consumption of high-sugar and trans-fatty acid foods could potentially prevent eczema development.

The findings of this research indicated that significant alterations in SIRI show an independent association with eczema prevalence in children and adolescents based on the NHANES database. Moreover, a non-linear relationship was shown between SIRI and eczema prevalence. This study represents the first investigation with a large sample examining the association between SIRI levels and eczema prevalence in pediatric and adolescent populations. Utilizing data from the nationally representative NHANES database strengthened the generalizability of our findings. The comprehensive adjustment for potential confounding variables enhanced the validity of the observed associations.

Several constraints also appear in this study. First, the diagnostic criteria for eczema in this study were limited to questionnaire-based assessments available in the NHANES database, which may lack diagnostic precision compared to comprehensive clinical evaluation. And potential confounding factors have not been ruled out, relevant studies have shown that local use of steroids (43), or serum vitamin D levels (44) can have an impact on SIRI-related parameters. Second, although this study accounted for various confounding variables, the conceivable implications of unintegrated covariates remain. Third, we did not conduct a multicollinearity test to rule out the mutual influence between covariates. Fourth, our analysis did not incorporate sampling weights, which could enhance the accuracy of the findings. Future studies that incorporate weights are likely to provide a better representation of the national situation. Moreover, the cross-sectional study is unable to demonstrate a cause-and-effect correlation between SIRI and eczema, highlighting the necessity for further investigation.

Our findings suggest several critical next steps. First, multi-omics analyses (e.g., transcriptomics, metabolomics) could be performed in patients with eczema exhibiting low SIRI to identify key signaling pathways. Second, a prospective cohort study may be adopted to verify whether low SIRI can predict the onset or recurrence of eczema. Third, randomized controlled trials (RCTs) could be conducted to monitor the dynamic changes in SIRI and their association with eczema severity. Finally, whether dietary interventions can improve eczema by elevating the SIRI index and modulating immune balance requires further clinical investigation.

## 5 Conclusion

The association between SIRI and eczema prevalence in children and adolescents is negative. The results indicate that elevated SIRI exhibits a protective effect against eczema in children and adolescents, whereas those with low SIRI may require closer monitoring for eczema development. Nonetheless, the current results are unable to demonstrate a cause-and-effect relationship between the factors, highlighting the necessity for further longitudinal investigations to corroborate the authors’ results.

## Data Availability

The original contributions presented in the study are included in the article/Supplementary Material, further inquiries can be directed to the corresponding author.

## References

[1] Murphrey MB Miao JH Zito PM. Histology, Stratum Corneum. Statpearls. Treasure Island (FL): StatPearls Publishing Copyright © 2025, StatPearls Publishing LLC (2025). Ineligible companies. Disclosure: Julia Miao declares no relevant financial relationships with ineligible companies. Disclosure: Patrick Zito declares no relevant financial relationships with ineligible companies. 30020671

[2] Weidinger S Novak N. Atopic dermatitis. Lancet. (2016) 387(10023):1109–22. 10.1016/s0140-6736(15)00149-x 26377142

[3] Torres T Ferreira EO Gonçalo M Mendes-Bastos P Selores M Filipe P. Update on atopic dermatitis. Acta Med Port. (2019) 32(9):606–13. 10.20344/amp.11963 31493365

[4] Al Nahas S Abouammoh N Althagafi W Alomary SA Almutairi AS Assiri AM Prevalence, severity, and risk factors of eczema among young children and adolescents in Saudi Arabia: a national cross-sectional study, 2019. J Allergy Clin Immunol Glob. (2024) 3(4):100299. 10.1016/j.jacig.2024.100299 39170912 PMC11338081

[5] García-Marcos L Asher MI Pearce N Ellwood E Bissell K Chiang CY The burden of asthma, hay fever, and eczema in children in 25 countries: gan phase I study. Eur Respir J. (2022) 60(3):2102866. 10.1183/13993003.02866-2021 35144987 PMC9474895

[6] Kahremany S Hofmann L Harari M Gruzman A Cohen G. Pruritus in psoriasis and atopic dermatitis: current treatments and new perspectives. Pharmacological Reports: PR. (2021) 73(2):443–53. 10.1007/s43440-020-00206-y 33460006

[7] Schonmann Y Mansfield KE Hayes JF Abuabara K Roberts A Smeeth L Atopic eczema in adulthood and risk of depression and anxiety: a population-based cohort study. J Allergy Clin Immunol Pract. (2020) 8(1):248–57.e16. 10.1016/j.jaip.2019.08.030 31479767 PMC6947493

[8] Drucker AM Wang AR Li WQ Sevetson E Block JK Qureshi AA. The burden of atopic dermatitis: summary of a report for the national eczema association. J Invest Dermatol. (2017) 137(1):26–30. 10.1016/j.jid.2016.07.012 27616422

[9] Linnet J Jemec GB. An assessment of anxiety and dermatology life quality in patients with atopic dermatitis. Br J Dermatol. (1999) 140(2):268–72. 10.1046/j.1365-2133.1999.02661.x 10233221

[10] Cheng CM Hsu JW Huang KL Bai YM Su TP Li CT Risk of developing major depressive disorder and anxiety disorders among adolescents and adults with atopic dermatitis: a nationwide longitudinal study. J Affect Disord. (2015) 178:60–5. 10.1016/j.jad.2015.02.025 25795537

[11] Kiebert G Sorensen SV Revicki D Fagan SC Doyle JJ Cohen J Atopic dermatitis is associated with a decrement in health-related quality of life. Int J Dermatol. (2002) 41(3):151–8. 10.1046/j.1365-4362.2002.01436.x 12010340

[12] Talamonti M Galluzzo M Silvaggio D Lombardo P Tartaglia C Bianchi L. Quality of life and psychological impact in patients with atopic dermatitis. J Clin Med. (2021) 10(6):1298. 10.3390/jcm10061298 33801061 PMC8003909

[13] Su JC Kemp AS Varigos GA Nolan TM. Atopic eczema: its impact on the family and financial cost. Arch Dis Child. (1997) 76(2):159–62. 10.1136/adc.76.2.159 9068310 PMC1717083

[14] Li H Zhang Z Zhang H Guo Y Yao Z. Update on the pathogenesis and therapy of atopic dermatitis. Clin Rev Allergy Immunol. (2021) 61(3):324–38. 10.1007/s12016-021-08880-3 34338977

[15] Kezic S Novak N Jakasa I Jungersted JM Simon M Brandner JM Skin barrier in atopic dermatitis. Front Biosci (Landmark Ed). (2014) 19(3):542–56. 10.2741/4225 24389202

[16] Leung DY. New insights into atopic dermatitis: role of skin barrier and immune dysregulation. Allergol Int. (2013) 62(2):151–61. 10.2332/allergolint.13-RAI-0564 23712284 PMC8609663

[17] Tokura Y Yunoki M Kondo S Otsuka M. What is “Eczema"? J Dermatol. (2025) 52(2):192–203. 10.1111/1346-8138.17439 39301836 PMC11807370

[18] Tay ASL Li C Nandi T Chng KR Andiappan AK Mettu VS Atopic dermatitis microbiomes stratify into ecologic dermotypes enabling microbial virulence and disease severity. J Allergy Clin Immunol. (2021) 147(4):1329–40. 10.1016/j.jaci.2020.09.031 33039480

[19] Geoghegan JA Irvine AD Foster TJ. Staphylococcus aureus and atopic dermatitis: a complex and evolving relationship. Trends Microbiol. (2018) 26(6):484–97. 10.1016/j.tim.2017.11.008 29233606

[20] Narla S Silverberg JI. The role of environmental exposures in atopic dermatitis. Curr Allergy Asthma Rep. (2020) 20(12):74. 10.1007/s11882-020-00971-z 33047271

[21] Morar N Willis-Owen SA Moffatt MF Cookson WO. The genetics of atopic dermatitis. J Allergy Clin Immunol. (2006) 118(1):24–34; quiz 5–6. 10.1016/j.jaci.2006.03.037 16815134

[22] Haybar H Pezeshki SMS Saki N. Evaluation of complete blood count parameters in cardiovascular diseases: an early indicator of prognosis? Exp Mol Pathol. (2019) 110:104267. 10.1016/j.yexmp.2019.104267 31194963

[23] Xia Y Xia C Wu L Li Z Li H Zhang J. Systemic immune inflammation index (SII), system inflammation response index (SIRI) and risk of all-cause mortality and cardiovascular mortality: a 20-year follow-up cohort study of 42,875 US adults. J Clin Med. (2023) 12(3):1128. 10.3390/jcm12031128 36769776 PMC9918056

[24] Hu B Yang XR Xu Y Sun YF Sun C Guo W Systemic immune-inflammation index predicts prognosis of patients after curative resection for hepatocellular carcinoma. Clin Cancer Res. (2014) 20(23):6212–22. 10.1158/1078-0432.Ccr-14-0442 25271081

[25] Qi Q Zhuang L Shen Y Geng Y Yu S Chen H A novel systemic inflammation response index (Siri) for predicting the survival of patients with pancreatic cancer after chemotherapy. Cancer. (2016) 122(14):2158–67. 10.1002/cncr.30057 27152949

[26] Dziedzic EA Gąsior JS Tuzimek A Paleczny J Junka A Dąbrowski M Investigation of the associations of novel inflammatory biomarkers-systemic inflammatory index (SII) and systemic inflammatory response index (SIRI)-with the severity of coronary artery disease and acute coronary syndrome occurrence. Int J Mol Sci. (2022) 23(17):9553. 10.3390/ijms23179553 36076952 PMC9455822

[27] Yang J Gong L Zhu X Wang Y Li C. Mediation of systemic inflammation response index in the association of healthy eating index-2020 in patientis with periodontitis. Oral Health Prev Dent. (2025) 23:225–32. 10.3290/j.ohpd.c_1946 40231719 PMC12089967

[28] Liu J Li G Wu R Qin X Pan S Liang P The systemic inflammatory response Index as a novel diagnostic indicator for Bell’s palsy. Br J Hosp Med. (2024) 85:1–14. 10.12968/hmed.2024.0386 39347675

[29] Zhang S Cheng T. Prognostic and clinicopathological value of systemic inflammation response index (SIRI) in patients with breast cancer: a meta-analysis. Ann Med. (2024) 56(1):2337729. 10.1080/07853890.2024.2337729 38569199 PMC10993763

[30] Zhang W-H Zhao Y Zhang C-R Huang J-C Lyu S-C Lang R. Preoperative systemic inflammatory response index as a prognostic marker for distal cholangiocarcinoma after pancreatoduodenectomy. World J Gastrointest Surg. (2024) 16(9):2910–24. 10.4240/wjgs.v16.i9.2910 39351557 PMC11438816

[31] Zhang D Zeng Y Sun B Li W Liu W Gao H Inflammatory indices—systemic immune-inflammation index (SII) and systemic inflammatory response Index (SIRI)—during pregnancy and associations with gestational diabetes mellitus. J Inflamm Res. (2024) 17:6521–32. 10.2147/jir.S474154 39310897 PMC11416769

[32] Cheng W Bu X Xu C Wen G Kong F Pan H Higher systemic immune-inflammation index and systemic inflammation response index levels are associated with stroke prevalence in the asthmatic population: a cross-sectional analysis of the NHANES 1999–2018. Front Immunol. (2023) 14:1191130. 10.3389/fimmu.2023.1191130 37600830 PMC10436559

[33] Guo M Zhu C. Serum neurofilament light chain, Markers of systemic inflammation and clinically relevant depressive symptoms in US adults. J Affect Disord. (2024) 363:572–8. 10.1016/j.jad.2024.07.146 39074516

[34] Nishida C Ko GT Kumanyika S. Body fat distribution and noncommunicable diseases in populations: overview of the 2008 WHO expert consultation on waist circumference and waist-hip ratio. Eur J Clin Nutr. (2010) 64(1):2–5. 10.1038/ejcn.2009.139 19935820

[35] Sun C Li XG Qian H Liang GR Xiang RY Zhao CJ Neutrophil-to-lymphocyte ratio and platelet-to-lymphocyte ratio are positively correlated with disease activity of bullous pemphigoid. Arch Dermatol Res. (2023) 315(8):2383–91. 10.1007/s00403-023-02639-w 37204459

[36] Tiucă OM Morariu SH Mariean CR Tiucă RA Nicolescu AC Cotoi OS. Impact of blood-count-derived inflammatory markers in psoriatic disease progression. Life. (2024) 14(1):114. 10.3390/life14010114 38255729 PMC10820213

[37] Ma R Cui L Cai J Yang N Wang Y Chen Q Association between systemic immune inflammation index, systemic inflammation response index and adult psoriasis: evidence from NHANES. Front Immunol. (2024) 15:1323174. 10.3389/fimmu.2024.1323174 38415255 PMC10896999

[38] Morariu S-H Cotoi OS Tiucă OM Baican A Gheucă-Solovăstru L Decean H Blood-count-derived inflammatory markers as predictors of response to biologics and small-molecule inhibitors in psoriasis: a multicenter study. J Clin Med. (2024) 13(14):3992. 10.3390/jcm13143992 39064032 PMC11277525

[39] Quaade AS Litman T Wang X Becker C McCauley BD Sølberg JBK Transcriptomic profiling of chronic hand eczema skin reveals shared immune pathways and molecular drivers across subtypes. J Allergy Clin Immunol. (2025) 155(4):1250–63. 10.1016/j.jaci.2024.12.1091 39793713

[40] Horiuchi Y. Th1 regulatory events by infectious pathogens, herpes zoster and herpes simplex viruses: prospects for therapeutic options for atopic eczema. Postepy Dermatol Alergol. (2022) 39(4):662–7. 10.5114/ada.2022.118920 36090727 PMC9454353

[41] Facheris P Da Rosa JC Pagan AD Angelov M Del Duca E Rabinowitz G Age of onset defines two distinct profiles of atopic dermatitis in adults. Allergy. (2023) 78(8):2202–14. 10.1111/all.15741 37032461

[42] Frischmeyer-Guerrerio PA Guerrerio AL Oswald G Chichester K Myers L Halushka MK Tgfβ receptor mutations impose a strong predisposition for human allergic disease. Sci Transl Med. (2013) 5(195):195ra94. 10.1126/scitranslmed.3006448 23884466 PMC3905327

[43] Muller I Stuart B Sach T Yardley L Greenwell K Becque T Programme Grants for Applied Research. Supporting Self-Care for Eczema in the Community: The Eczema Care Online Research Programme Including Two RCTs. Southampton (UK): National Institute for Health and Care Research Copyright © 2025 Muller et al. (2025). 40168498

[44] Wei J Jaleel T MacLeod AS Ji JS. Inverted U-shaped relationship between vitamin D and ever-reported eczema in US adults. Allergy. (2019) 74(5):964–75. 10.1111/all.13708 30589434

